# DNA repair prognostic index modelling reveals an essential role for base excision repair in influencing clinical outcomes in ER negative and triple negative breast cancers

**DOI:** 10.18632/oncotarget.4157

**Published:** 2015-06-01

**Authors:** Tarek M.A. Abdel-Fatah, Arvind Arora, Paul M. Moseley, Christina Perry, Emad A. Rakha, Andrew R. Green, Stephen Y.T. Chan, Ian O. Ellis, Srinivasan Madhusudan

**Affiliations:** ^1^ Department of Oncology, Nottingham University Hospitals, Nottingham NG5 1PB, UK; ^2^ Academic Unit of Oncology, Division of Cancer and Stem Cells, School of Medicine, University of Nottingham, Nottingham NG51 PB, UK; ^3^ Department of Pathology, Division of Cancer and Stem Cells, School of Medicine, University of Nottingham, Nottingham NG5 1PB, UK

**Keywords:** DNA repair, base excision repair, ER-, TNBC, prognosis

## Abstract

Stratification of oestrogen receptor (ER) negative and triple negative breast cancers (TNBCs) is urgently needed. In the current study, a cohort of 880 ER- (including 635 TNBCs) was immuno-profiled for a panel of DNA repair proteins including: Pol β, FEN1, APE1, XRCC1, SMUG1, PARP1, BRCA1, ATR, ATM, DNA-PKcs, Chk1, Chk2, p53, and TOPO2. Multivariate Cox proportional hazards models (with backward stepwise exclusion of these factors, using a criterion of *p* < 0.05 for retention of factors in the model) were used to identify factors that were independently associated with clinical outcomes. XRCC1 (*p* = 0.002), pol β (*p* = 0.032) FEN1 (*p* = 0.001) and BRCA1 (*p* = 0.040) levels were independently associated with poor BCSS. Subsequently, DNA repair index prognostic (DRPI) scores for breast cancer specific survival (BCSS) were calculated and two prognostic groups (DRPI-PGs) were identified. Patients in prognostic group 2 (DRPI-PG2) have higher risk of death (*p* < 0.001). Furthermore, in DRPI-PG2 patients, exposure to anthracycline reduced the risk of death [(HR (95% CI) = 0.79 (0.64–0.98), *p* = 0.032) by 21–26%. In addition, DRPI-PG2 patients have adverse clinicopathological features including higher grade, lympho-vascular invasion, Her-2 positive phenotype, compared to those in DRPI-PG1 (*p* < 0.01). Receiver operating characteristic (ROC) curves indicated that the DRPI outperformed the currently used prognostic factors and adding DRPI to lymph node stage significantly improved their performance as a predictor for BCSS [*p* < 0.00001, area under curve (AUC) = 0.70]. BER strongly influences pathogenesis of ER- and TNBCs. The DRPI accurately predicts BCSS and can also serve as a valuable prognostic and predictive tool for TNBCs.

## INTRODUCTION

About 15%–20% of breast cancers (BC) are triple negative BC (TNBC) subtype which is characterized by aggressive phenotype with rapid proliferation, invasion, metastasis and poor survival [1–3]. Treatment strategies for patients with ER negative (ER-) and TNBC are limited [4] and remains a significant clinical challenge, hindered by the inability of these tumours to respond to traditional hormone therapies and targeted agents. Research efforts are urgently needed to discover specific prognostic and predictive molecular signatures that can guide individualized therapy for this BC subgroup [5]. Although recently published gene expression data revealed that TNBC is largely heterogeneous, impaired DNA repair machinery is a common feature of different TNBC molecular subtypes [6, 7]. Moreover, recent studies suggest that the clinical outcome and response to current standard chemotherapy in ER-BCs, especially TNBC, are particularly influenced by the integrity of DNA repair pathways [7]. The higher rate of response to a given chemotherapy could probably result from an accumulation of DNA damage, abnormal mitoses and subsequent mitotic catastrophe [5].

Given the complex network of DNA repair machinery, we speculated that in ER- and TNBCs deregulation of multiple DNA damage signalling and DNA repair pathways could have a significant impact on prognosis and response to therapy. To address this hypothesis, we immunohistochemically-profiled a panel of key factors involved in DNA-damage signalling network (ATR, ATM, pChk1, Chk2, p53), double strand break repair (BRCA1, DNA-PKcs, TOPO2) and base excision repair (pol β, FEN1, APE1, XRCC1, SMUG1, PARP1) in a large cohort of 880 ER-breast cancers (including 635 TNBCs) with long term follow-up data. We demonstrated that BER (XRCC1, polβ and FEN1) independently influenced clinical outcome along with BRCA1. A DNA repair prognostic index incorporating XRCC1, polβ, FEN1 and BRCA1 stratified patients into two distinct prognostic groups. The primary potential clinical significance of our results is the ability to identify those patients with ER- or TNBC who are likely to benefit from the standard adjuvant anthracycline chemotherapy, and spare other patients whose response would be poor from enduring the unnecessary serious cytotoxic side-effects.

## RESULTS

### Deregulated XRCC1, pol β, FEN1 and BRCA1 are independently associated with poor survival in ER negative breast cancers

Clinico-pathological factors associated with BCSS in univariate Cox analysis included pol β, FEN1, APE1, XRCC1, SMUG1, PARP1, ATR, ATM, pChk1, Chk2, p53, BRCA1, DNA-PKcs, TOPO2, Bcl-2, androgen receptor (AR), tumour stage, histological grade, tumour size, lymph-vascular invasion and lymph node status. By using multivariate Cox proportional hazards models with backward stepwise exclusion, XRCC1 (*p* = 0.002), FEN1 (*p* = 0.001), pol β (*p* = 0.032), BRCA1 (*p* = 0.040) and tumour stage (*p* < 0.0001) remained significant independent predictors for BCSS after controlling for adjuvant chemotherapy (Table 1). None of the covariates exhibited significant deviations from the proportionality assumption or had time-dependent effects)

**Table 1:** Multivariate Cox proportional hazards model in ER negative breast cancers

Variables	Beta	*p*-value	Risk ratio	Risk ratio 95% lower	Risk ratio 95% upper
XRCC1	−.214	**0.002**	.807	0.706	0.923
Pol β	−.226	**0.032**	.797	0.648	0.981
FEN1	.391	**0.001**	1.479	1.175	1.861
BRCA1	−.473	**0.040**	.623	0.397	0.979
Tumour Stage	1.001	**0.000**	2.722	2.108	3.515

### Development of DNA repair prognostic index (DRPI) for BCSS

Subsequently, the summations of β coefficient values of XRCC1, FEN1, pol β and BRCA1 in the final Cox model were used to calculate the DRPI for each patient as follows:

DRPI score = XRCC1 (high; −0.214, low; 0) + FEN1 (high; 0.391, low; 0) + polβ (high; −0.226, low; 0) + BRCA1 (high; −0.473, low; 0)

### DRPI stratifies patients into two distinct prognostic groups (DRPI-PGs)

Based on the cut-off point selected at the quantile of DRPI score that maximized the profile log-likelihood of the model, two DRPI-PGs were identified. DRPI-PG1 was defined as tumours with DRPI score ranging from −0.31 to −0.91 while DRPI-PG2 was defined as tumours with DRPI score ranging from 0.39 to −0.30.

**Figure 1:**
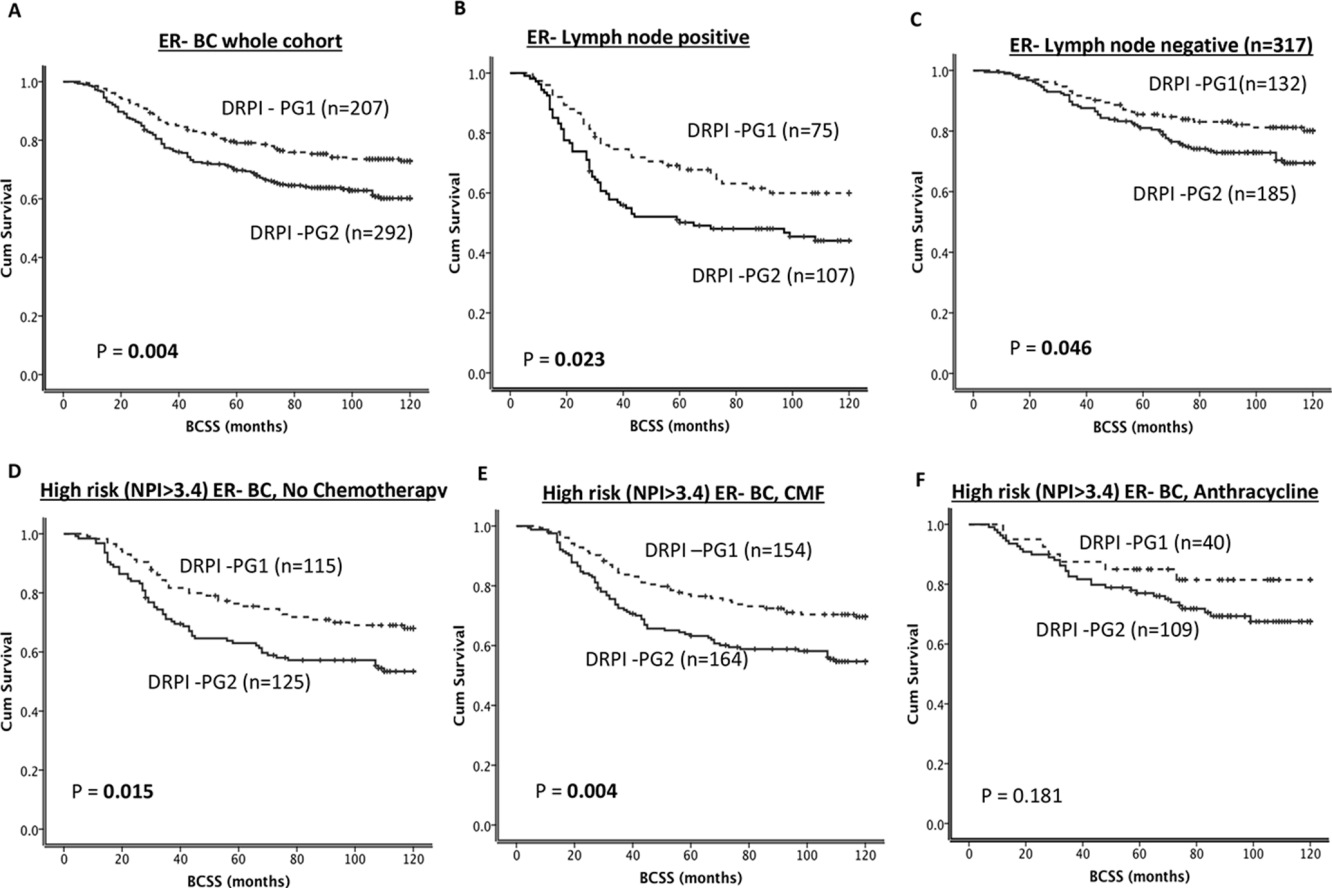
DRPI and survival **A.** Kaplan Meier curves showing BCSS based on DRPI groups in ER- patients **B.** Kaplan Meier curves showing BCSS based on DRPI groups in ER-/lymph node positive patients **C.** Kaplan Meier curves showing BCSS based on DRPI groups in ER-/lymph node negative patients **D.** Kaplan Meier curves showing BCSS based on DRPI groups in ER- patients who received no chemotherapy. **E.** Kaplan Meier curves showing BCSS based on DRPI groups in ER- patients who received CMF chemotherapy. **F.** Kaplan Meier curves showing BCSS based on DRPI groups in ER- patients who received anthracycline chemotherapy.

**Figure 2:**
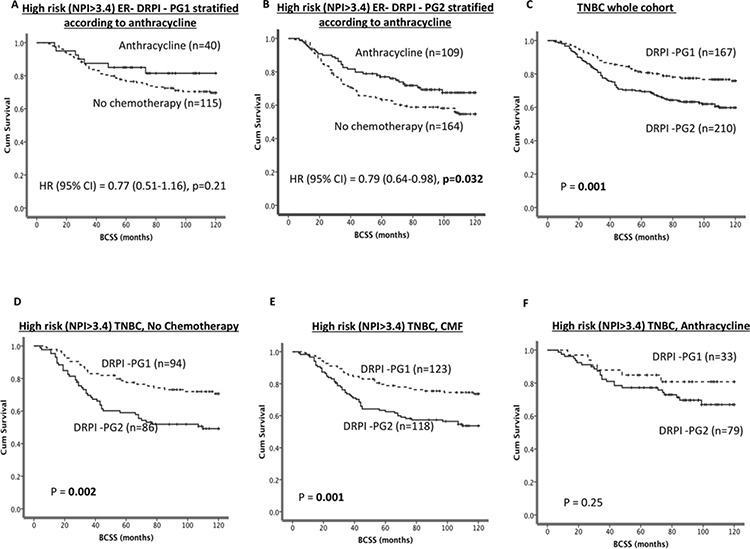
DRPI and survival **A.** Kaplan Meier curves showing BCSS in ER-/DRPI-PG1 stratified according to anthracycline. **B.** Kaplan Meier curves showing BCSS in ER-/DRPI-PG2 stratified according to anthracycline. **C.** Kaplan Meier curves showing BCSS based on DRPI groups in TNBC patients **D.** Kaplan Meier curves showing BCSS based on DRPI groups in TNBC patients who received no chemotherapy. **E.** Kaplan Meier curves showing BCSS based on DRPI groups in TNBC patients who received CMF chemotherapy. **F.** Kaplan Meier curves showing BCSS based on DRPI groups in TNBC patients who received anthracycline chemotherapy.

As shown in Figure 1A, patients belonging to DRPI -PG2 have poor BCSS compared to DRPI-PG1 (*p* = 0.004). In tumours with lymph node positivity, DRPI-PG2 remains associated with poor survival compared DRPI–PG1 (Figure 1B) (*p* = 0.023). Interestingly, even in lymph node negative tumours, DRPI-PG2 remains associated with poor survival compared to DRPI-PG1 (Figure 1C) (*p* = 0.046). In tumours that did not receive any chemotherapy or that received ineffective CMF chemotherapy, DRPI-PG2 remained associated with poor survival compared to DRPI-PG1 (Figure 1D, 1E) (ps = 0.015 and 0.004, respectively). In patients who received adjuvant anthracycline chemotherapy, tumours with DRPI-PG1 had a clinical outcome similar to those with DRPI-PG2 (*p* = 0.181, Figure 1F). No benefit was demonstrated from prescribing an anthracycline in DRPI-PG1 (Figure 2A), whereas for DRPI-PG2, exposure to an anthracycline reduced the risk of death from BC by 21–26% [(HR (95% CI) = 0.79 (0.64–0.98), *p* = 0.032) (Figure 2B); the interaction was statistically significant with *p* = 0.04.

### DRPI-PGs in triple negative breast cancers (TNBC)

**Figure 3:**
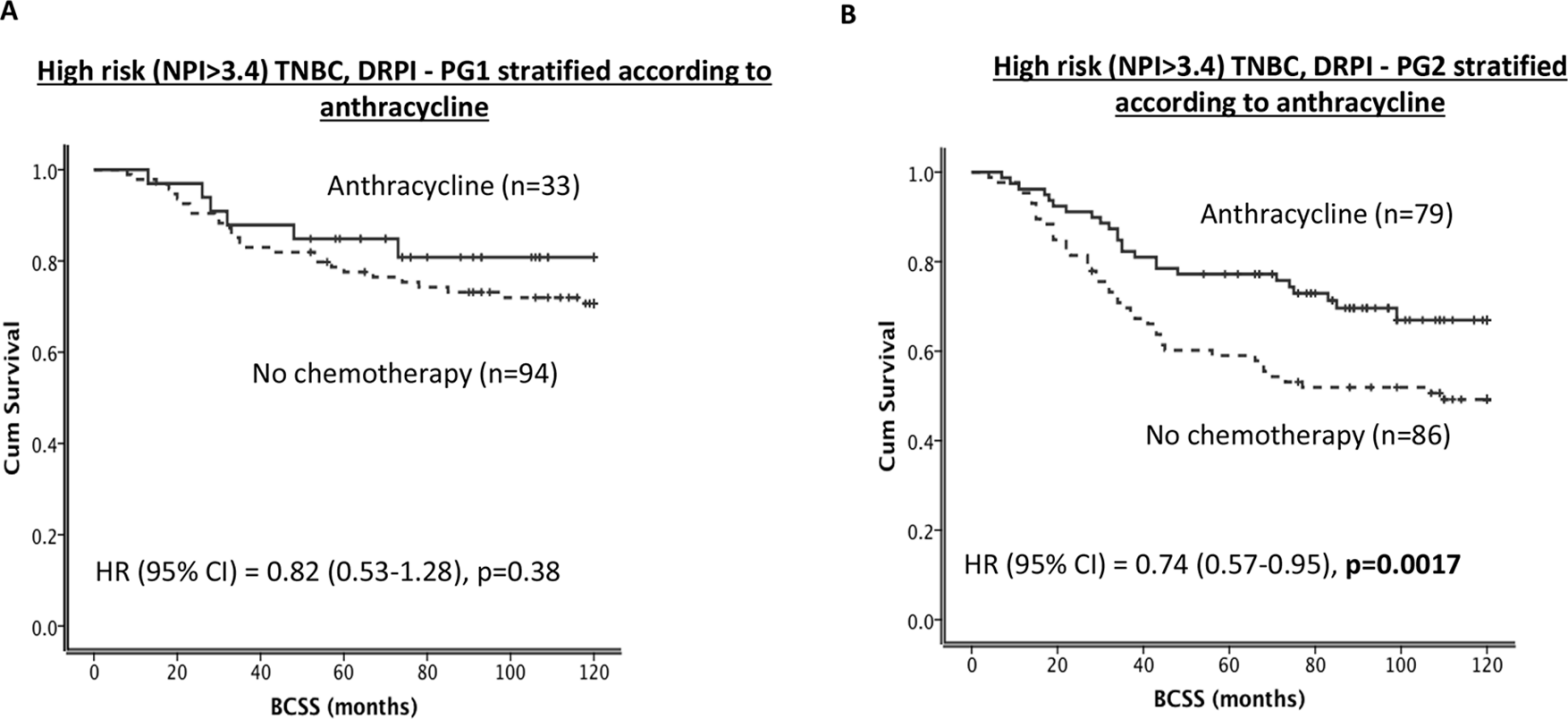
DRPI and survival **A.** Kaplan Meier curves showing BCSS in TNBC/DRPI-PG1 stratified according to anthracycline. **B.** Kaplan Meier curves showing BCSS in TNBC/DRPI-PG2 stratified according to anthracycline.

As shown in Figure 2C, patients with tumours in DRPI-PG2 have poor BCSS compared to DRPI-PG1 (*p* = 0.001). In patients who either did not receive any chemotherapy or received ineffective adjuvant CMF chemotherapy, DRPI-PG2 remains associated with poor survival compared to DRPI-PG1 (Figure 2D and 2E, *p* = 0.002 and *p* = 0.001, respectively). On the other hand, in patients who received adjuvant anthracycline chemotherapy, there was no significant association (*p* = 0.25, Figure 2F). For patients with DRPI-PG2 exposure to anthracycline reduced the risk of death from TNBC (HR (95% CI) = 0.74 (0.57–0.95), *p* = 0.0017) by 26% (Figure 3B) while, no benefit was demonstrated with anthracycline in DRPI-PG1 (Figure 3A); the interaction was statistically significant with *p* = 0.001.

**Figure 4:**
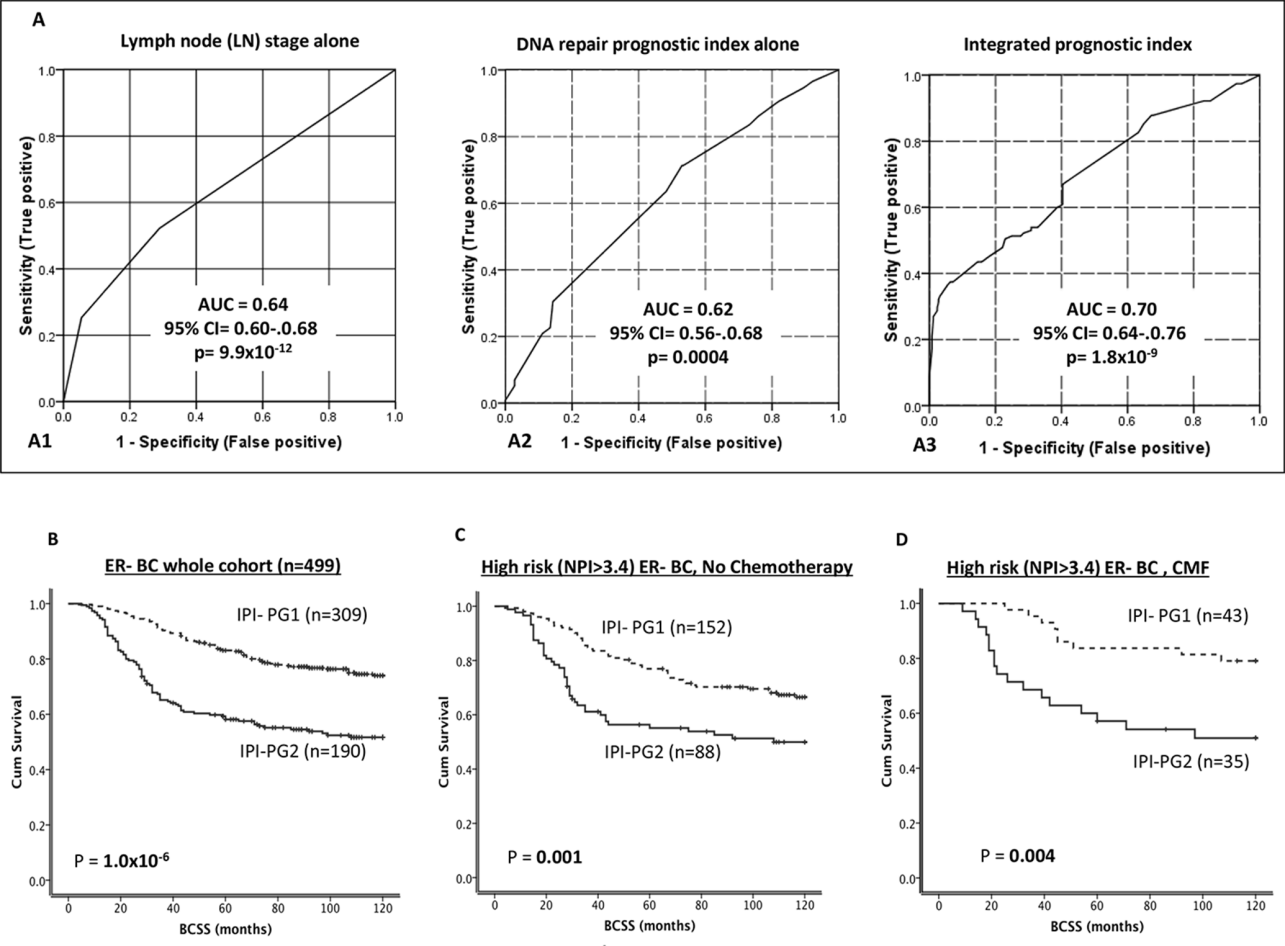
**A. Receiver operating characteristic (ROC) curves [A1. Lymphnode stage only, A2. DRPI only, A3. Integrated prognostic index (IPI)]**. **B.** Kaplan Meier curves showing BCSS based on IPI groups in ER- patients. **C.** Kaplan Meier curves showing BCSS based on IPI groups in ER- patients who received no chemotherapy. **D.** Kaplan Meier curves showing BCSS based on IPI groups in ER- patients who received CMF chemotherapy.

To examine the prognostic performance of the DRPI score compared with other prognostic factors such lymph node stage, Cox proportional hazards multivariable analysis after controlling for adjuvant chemotherapy was performed and receiver operating characteristic (ROC) curves were generated. DRPI score and LN stages were the only independent prognostic factors for ER- BC (ps < 0.001). Furthermore as shown in Figures 4A1 and 4A2, the ROC curves demonstrate that the AUC for lymph node stage and DRPI are similar (AUCs 0.64 and 0.62 respectively); however, adding DRPI to LN stage improved their performance as a prognostic tool for BCSS (AU*C* = 0.70), Figure 4A3.

Taken together the data suggest that DRPI has both prognostic and predictive significance in early stage ER- BC and TNBC breast cancers.

### DRPI-PGs and clinicopathological associations

As shown in Table 2, tumours in DRPI-PG2 were associated with adverse clinicopathological features including higher histologic grade (*p* = 0.045), lympho-vascular invasion (*p* = 0.001), and Her-2 overexpression (*p* = 0.046). Furthermore, DRPI-PG2 was more likely to have low levels of other DNA repair proteins including: PARP1 (*p* = 0.002), ATM level (*p* = 0.018), nuclear pChk1 (*p* = 0.001), Chk2 level (*p* < 0.001), and APE1 levels (*p* < 0.001). Interestingly, DRPI-PG1 tumours were more likely to be associated with basal-like phenotype (*p* = 0.024), high proliferation markers such as Ki67 (*p* = 0.032) and Top2A overexpression (*p* = 0.004) and epithelial-mesenchymal transition markers such as CK5/6 (*p* = 0.006), E-cadherin (*p* < 0.001) and P-cadherin (*p* = 0.018) levels.

**Table 2:** DNA repair prognostic index (DRPI) and ER- breast cancer

VARIABLE	DNA Repair Prognostic Index (DRPI)		*P*-value
	DRPI-PG1N (%)	DRPI-PG2N (%)	
**A) Pathological Parameters**			
**Tumour Size** < 1 cm > 1–2 cm > 2–5 cm > 5 cm	14 (6.8) 98 (47.6) 88 (42.7) 6 (2.9)	23 (8.0) 122 (42.4) 131 (45.5) 12 (4.2)	0.640
**Tumour Stage** 1 2 3	134 (63.5) 51 (24.2) 26 (12.3)	185 (63.1) 69 (23.5) 39 (13.3)	0.944
**Tumour Grade** G1 G2 G3	4 (1.9) 15 (7.1) 192 (91.0)	0 (0.0) 27 (9.2) 266 (90.8)	**0.045**
**Mitotic Index** M1 (low; mitoses < 10) M2 (medium; mitoses 10–18) M3 (high; mitosis > 18)	12 (5.7) 23 (10.9) 176 (83.4)	16 (5.5) 37 (12.7) 238 (81.8)	0.825
**Tubule Formation** 1 (> 75% of definite tubule) 2 (10% − 75% definite tubule) 3 (< 10% definite tubule)	1 (0.5) 30 (14.2) 180 (85.3)	1 (0.3) 47 (16.2) 243 (83.5)	0.820
**Tumour Type** IDC-NST Medullary Carcinoma Tubular Carcinoma ILC Others	176 (87.6) 9 (4.5) 2 (1.0) 8 (4.0) 6 (3.0)	257 (88.9) 14 (4.8) 5 (1.7) 4 (1.4) 9 (3.1)	0.437
**Lymphovascular Invasion** No Yes	110 (52.9) 98 (47.1)	120 (41.2) 171 (58.8)	**0.010**
**Molecular sub-types** Non Luminal HER2 over expression2 Basal Like ER-/HER2-	38 (19.3) 127 (64.5) 32 (16.2)	74 (26.3) 152 (54.1) 55 (19.6)	0.071
**B) Aggressive phenotype**			
**Her2 overexpression** No Yes	173 (82.0) 38 (18.0)	216 (74.5) 74 (25.5)	**0.046**
**Triple Negative Phenotype** No Yes	40 (19.0) 170 (81.0)	76 (26.5) 211 (73.5)	0.053
**Basal Like Phenotype** No Yes	70 (35.5) 127 (64.5)	129 (45.9) 152 (54.1)	**0.024**
**Cytokeratin 6 (CK6)** Negative Positive	101 (52.3) 92 (47.7)	178 (65.0) 96 (35.0)	**0.006**
**Cytokeratin 14 (CK14)** Negative Positive	145 (74.7) 49 (25.3)	205 (75.4) 67 (24.6)	0.878
**Cytokeratin 18 (CK18)** Negative Positive	97 (50.5) 95 (49.5)	139 (53.1) 123 (46.9)	0.594
**Cytokeratin 19 (CK19)** Negative Positive	16 (13.8) 100 (86.2)	14 (13.2) 92 (86.8)	0.899
**E-cadherin** Low Overexpression	40 (20.6) 154 (79.4)	99 (37.5) 165 (62.5)	**1.0 × 10^−4^**
**P-cadherin** Low Overexpression	22 (11.7) 166 (88.3)	54 (20.1) 215 (79.9)	**0.018**
**C) Hormone receptors**			
**PgR** Negative Positive	201 (99.5) 1 (0.5)	267 (98.2) 5 (1.8)	0.196
**AR** Negative Positive	85 (76.6) 26 (23.4)	69 (69.7) 30 (30.3)	0.260
**D) DNA Repair**			
**PARP1** Low High	107 (61.1) 68 (38.9)	196 (75.1) 65 (24.9)	**0.002**
**SMUG1** Low High	69 (40.8) 100 (59.2)	97 (36.7) 167 (63.3)	0.394
**APE1** Low High	50 (46.7) 57 (53.3)	67 (72.8) 25 (27.2)	**1.9 × 10^−4^**
**ATR** Low High	71 (39.9) 107 (60.1)	94 (38.1) 153 (61.9)	0.702
**ATM** Low High	90 (55.9) 71 (44.1)	157 (67.7) 75 (32.3)	**0.018**
**DNA-PKcs** Low High	36 (37.1) 61 (62.9)	43 (50.6) 42 (49.4)	0.067
**E) Cell cycle/apoptosis regulators**			
**P16** Low High	61 (57.5) 45 (42.5)	59 (64.8) 32 (35.2)	0.296
**P21** Low High	136 (74.3) 47 (25.7)	192 (73.3) 70 (26.7)	0.807
**MIB1** Low High	30 (26.8) 82(73.2)	47 (40.2) 70 (59.8)	**0.032**
**P53** Low expression High expression	75 (38.7) 119 (61.3)	114 (40.4) 168 (59.6)	0.699
**Bcl-2** Negative Positive	147 (73.1) 54 (26.9)	220 (78.3) 61 (21.7)	0.190
**TOP2A** Low Overexpression	82 (45.6) 98 (54.4)	155 (59.4) 106 (40.6)	**0.004**
**pChk1 (Nuclear)** Low High	180 (89.1) 22 (10.9)	256 (96.6) 9 (3.4)	**0.001**
**pChk1 (Cytoplasmic)** Low High	53 (26.2) 149 (73.8)	49 (18.5) 216 (81.5)	**0.045**
**Chk2** Low High	78 (45.9) 92 (54.1)	152 (63.9) 86 (36.1)	**3.0 × 10^−4^**
**Bax** Low High	65 (77.4) 19 (22.6)	56 (70.0) 24 (30.0)	0.283
**CDK1** Low High	55 (57.3) 41 (42.7)	47 (55.3) 38 (44.7)	0.787
**MDM2** Low Overexpression	93 (87.7) 13 (12.3)	75 (85.2) 13 (14.8)	0.610
**MDM4** Low Overexpression	127 (88.2) 17 (11.8)	193 (95.1) 10 (4.9)	**0.018**

*Statistically significant

**grade as defined by NGS; BRCA1: Breast cancer 1, early onset; HER2: human epidermal growth factor receptor 2; ER: oestrogen receptor; PgR: progesterone receptor; CK: cytokeratin; Basal-like: ER-, HER2 and positive expression of either CK5/6, CK14 or EGFR; Triple negative: ER-/PgR-/HER2-.

### Integrated prognostic index (IPI) and IPI-PGs accurately predicts clinical outcome after adjuvant chemotherapy

To generate a continuous integrated prognostic index score (IPI; range= 3.39–0.09) for ER-BC the LN stage score (1–3) has been added to DRPI as described in the methods section. Subsequently, two IPI-PGs were identified; IPI-PG1 = 3.39 to 1.09 and IPI-PG2 = 1.0 to 0.09.

**Figure 5:**
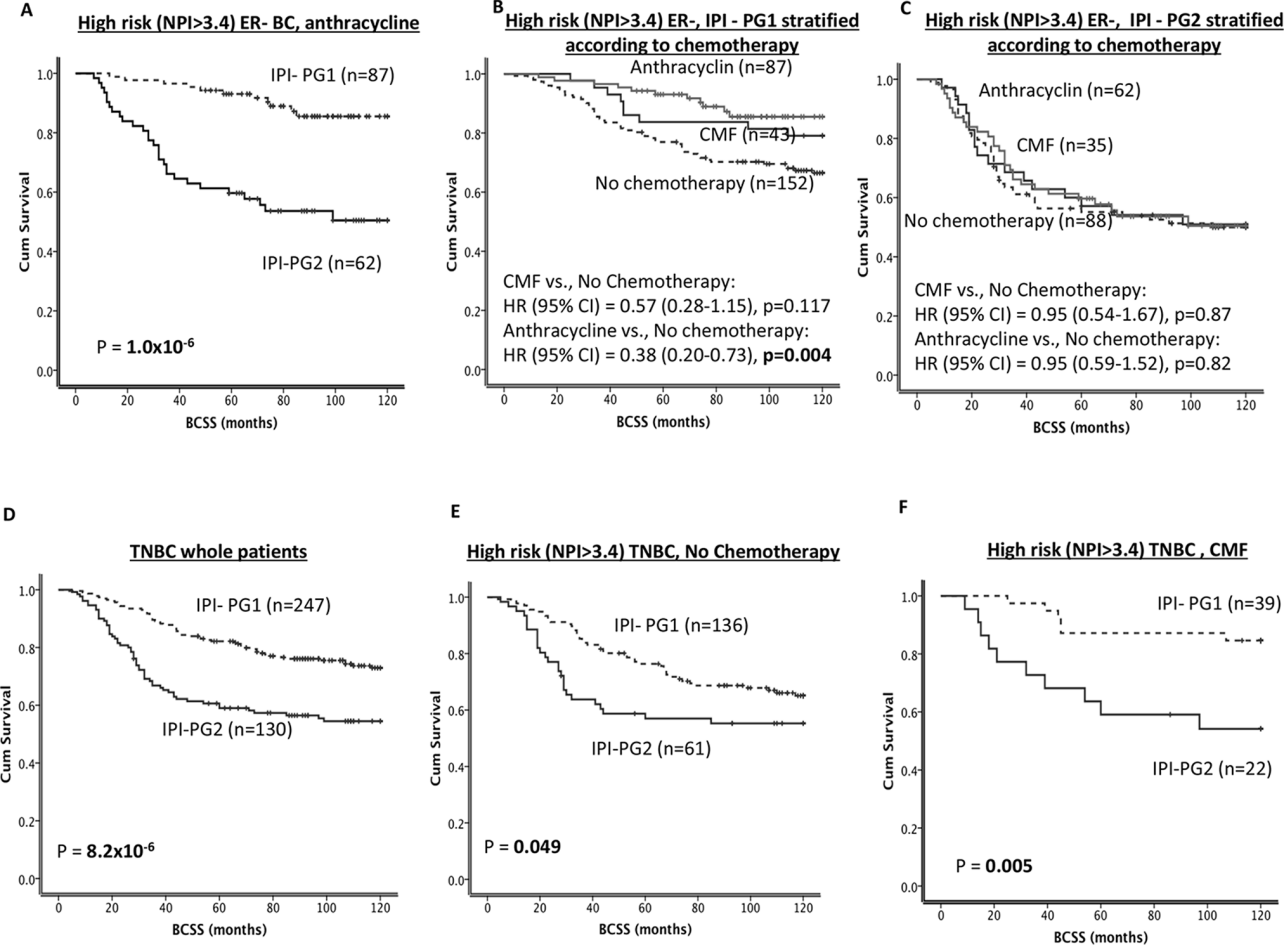
IPI and survival **A.** Kaplan Meier curves showing BCSS based on IPI groups in ER- patients who received anthracycline chemotherapy. **B**. Kaplan Meier curves showing BCSS in ER-/IPI-PG1 stratified according to chemotherapy. **C.** Kaplan Meier curves showing BCSS in ER-/IPI-PG2 stratified according to chemotherapy. **D.** Kaplan Meier curves showing BCSS based on IPI groups in TNBC patients. **E.** Kaplan Meier curves showing BCSS based on IPI groups in TNBC patients who received no chemotherapy. **F.** Kaplan Meier curves showing BCSS based on IPI groups in TNBC patients who received CMF chemotherapy.

In ER- negative tumours, BCSS was significantly poorer in IPI-PG2 compared to IPI-PG1 (*p* < 0.001) (Figure 4B). In patients who received no chemotherapy (Figure 4B) and ineffective CMF chemotherapy (Figure 4C), IPI-PG2 group have poor survival compared to IPI-PG1 group (*p* = 0.001 and *p* = 0.004 respectively). Similarly, in patients who received anthracycline chemotherapy, IPI-PG2 have poor survival compared to IPI-PG1 group (*p* < 0.001) (Figure 5A). Furthermore, for patients with IPI-PG1 exposure to anthracycline reduced the risk of death from BC (HR (95% CI) = 0.38 (0.20–0.73), *p* = 0.004) by 62% (Figure 5B), whereas for IPI sub-group 2, there was no effect (Figure 5C), the interaction was statistically significant at *p* = 0.007.

**Figure 6:**
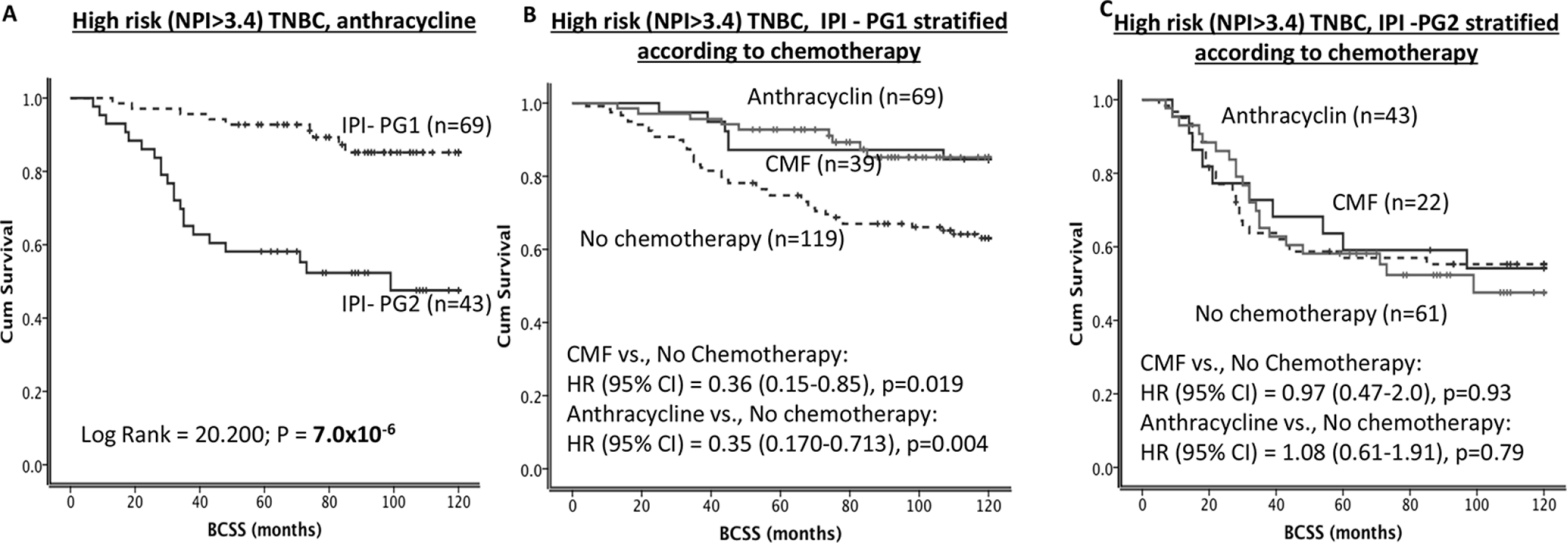
IPI and survival **A.** Kaplan Meier curves showing BCSS based on IPI groups in TNBC patients who received anthracycline chemotherapy. **B.** Kaplan Meier curves showing BCSS in TNBC/IPI-PG1 stratified according to chemotherapy. **C**. Kaplan Meier curves showing BCSS in TNBC/IPI-PG2 stratified according to chemotherapy.

Generally in TNBCs, BCSS was significantly poorer in IPI-PG2 compared to IPI-1 group (*p* < 0.0001) (Figure 5D). In TNBCs, patients who did not receive (*p* < 0.001; Figure 5E) or those received either CMF (*p* = 0.005; Figures 5E) or anthracycline (*p* < 0.001; Figure 6A) chemotherapies, BCSS was significantly poorer in IPI-PG2 group compared to IPI-PG1. However within IPI PG1 of TNBC, patients treated with either CMF or anthracycline had longer survival (*p* = 0.004) as compared to those who did not receive any chemotherapy. In TNBC patients with IPI-PG1 exposure to either CMF or anthracycline reduced the risk of death by 64% (HR (95% CI) = 0.36 (0.15–0.85), *p* = 0.019) and 65% (HR (95% CI) = 0.35 (0.170–0.713), *p* = 0.004), respectively (Figure 6B); the interaction was statistically significant with *p* = 0.001. No benefit was demonstrated for either CMF or anthracycline in IPI-PG2 (Figure 6C).

Taken together, the data provides compelling evidence that incorporation of DNA repair expression to lymph node status significantly improves prognostication and prediction of ER- and TNBC patients.

## DISCUSSION

This is a comprehensive immunohistochemical evaluation of the key DNA repair proteins in a large cohort of ER- and TNBC patients. We provide evidence that XRCC1, polβ and FEN1 independently impact outcomes and are superior to BRCA1 in prognosticating ER- or TNBC patients. The data presented here supports recent pre-clinical observations that implicate a cross talk between BER and BRCA1. BRCA1 has been shown to transcriptionally regulate the expression of BER factors such as OGG1, NTH1 and APE1 [8, 9]. Similarly in a recent study we consistently observed low expression of XRCC1 and polβ in BRCA1 deficient cell lines compared to BRCA1 proficient cell lines [10] implying a potential role for BRCA1 in regulating BER.

TNBC is characterized biologically by having a histopathological similarity with germline BRCA1-mutated breast cancer (BRCA-ness phenotype) [11–14] with 90% of BRCA1-mutation tumors being considered as TNBC [15]. Our study suggests that dysfunctional BRCA1 in TNBC could lead to impaired BER expression which in turn may promote aggressive clinical behaviours [16]. We speculate that increased genomic instability in BRCA1 deficient/BER impaired cells could promote a ‘mutator phenotype’ resulting in accelerated mutagenesis and aggressive biology [16]. In addition, recent studies implicate a role for BRCA1 in transcriptional regulation of nucleotide excision repair (NER) [17] and a role in NHEJ. BRCA1 is also well known to interact with DNA-damage signalling protein such as the ATM-Chk2 and ATR-Chk1 pathway that link DNA damage to repair, cell cycle progression and apoptosis [18–23]. In the current study, in both ER- and TNBC patients who either did not receive or received ineffective CMF based chemotherapy; survival was poor in the DRPI-PG2. Whereas patients in DRPI-PG2 who are more likely to have impairment of BER and DSB mediated DNA repair machinery exhibit relative sensitivity to anthracycline chemotherapy in ER-BC and TNBC as evidenced by improved survival in this group. Anthracycline chemotherapy induces oxidative base damage through free radical generation, which if unrepaired, could lead to the accumulation of DNA double strand breaks.

The main potential clinical significance of our results is the ability to identify patients with TNBC who are likely to benefit from the standard anthracycline chemotherapy, and spare patients whose response would be poor from enduring the unnecessary serious cytotoxic side effects. However, identification of patients who are unlikely to benefit from anthracycline chemotherapy on the bases of evaluation of DNA repair signature in the TNBC tumours either before initiation of Neoadjuvant chemotherapy- or shortly after surgery and before starting the additional adjuvant chemotherapy may have even a greater importance. Due to its deficiency of DNA repair mechanisms, BRCA1 mutation-associated TNBC cells are particularly sensitive to methyl methane sulfonate (alkylating agent) [24] and to DNA-damaging platinum agents, like cisplatin or carboplatin [25]. Recently, a phase II study evaluated cisplatin monotherapy as a neoadjuvant therapy in TNBC patients, showing a pCR rate of 22% [26]. For breast cancer patients with BRCA1 mutation, single-agent cisplatin neoadjuvant therapy can achieve an extremely high pCR rate of 83% [27]. Several phase II single-arm studies have tested the combination of taxane and platinum salts as neoadjuvant therapy for TNBC patients, with pCR rates of 33–77%, indicating that platinum salts are especially active in TNBC treatment [25]. Also BRCA1 mutated and basal-like breast cancer cells were found to be sensitive to oxidative DNA damage induced by H2O2 treatment [28]. The increased sensitivity was associated with defective BER as assessed by cell based BER assay in BRCA1 deficient cells [28]. Taken together, the data provides evidence that the DRPI is a promising predictive factor in ER- and TNBC patients.

In contrast to ER+ and HER-2 positive breast cancers, there is currently a lack of robust prognostic and predictive factors in ER- and TNBCs [1, 2]. Apart from lymph node stage [4], there is as yet no clinically validated biomarker in ER- and TNBCs. An important observation in the current study is that addition of DRPI appears to improve prognostic significance of lymph node stage as demonstrated by ROC analysis. To validate this further we combined DRPI scores and lymph node stage and generated an integrated prognostic index (IPI). As expected the IPI was robust in prognosticating as well as predicting outcomes in ER- and TNBC patients. To a large extent the data presented here is hypothesis generating and prospective validation will be required to translate our novel observations for patient benefit.

Gene expression profiling data suggest that TNBCs exhibit considerable heterogeneity [14]. Although genetic phenotypes provide biological insights, their clinical impact is uncertain. Therefore, a semi-quantitative immunohistochemical approach remains a clinically viable strategy. In this context our study complements the BRCAness phenotype described previously in TNBCs [13]. The promising synthetic lethality approach targeting the BRCAness phenotype in TNBCs with PARP inhibitors [17] suggest that additional DNA repair targets would also be suitable for personalized approaches. Interestingly, in preclinical models we have recently shown that BRCA1/BER deficient breast cancer cells are sensitive to treatment with ATM and DNA-PKcs treatment either alone or in combination with cisplatin chemotherapy [10] suggesting additional approaches besides PARP inhibitors for personalized strategy.

In conclusion we have shown that an immunohistochemical based DNA repair prognostic index could be utilized for stratification of clinical outcome in ER- and TNBC patients and can also serve as a valuable predictive tool for TNBCs. BER strongly influences pathogenesis of ER- and TNBCs.

## PATIENTS AND METHODS

We retrospectively identified 880 consecutive patients with ER negative early primary invasive BC who were diagnosed and treated in Nottingham University Hospitals, UK, between 1986 and 2010 and whose tissues were suitable for the analysis of a panel of key DNA repair proteins expression by immunohistochemistry. Patient demographics are summarized in additional file 1 (Supplementary Table S1). These patients were all female and their median age was 51 years (range 28–71 years). Their median follow-up was 107 months (range 2–243 months). Of these patients, 635 (72%) had triple negative phenotype (ER-, PR- and HER2-) and 185 (22%) were HER2 positive. Patients received standard breast surgery (mastectomy or wide local excision, and axillary clearance for node positive or sampling for node negative) with radiotherapy. Prior to 1989, patients did not receive adjuvant chemotherapy. Since 1989, adjuvant therapy was scheduled on the basis of the Nottingham Prognostic Index (NPI), ER-α and menopausal status [5]. Patients with NPI scores < 3.4 (low risk) did not receive adjuvant chemotherapy. Pre-menopausal patients with NPI scores ≥ 3.4 (high risk) were recommended to receive adjuvant chemotherapy. No adjuvant chemotherapy was prescribed to 503 cases, either because the patient declined the treatment; ACT was not the standard of care at that time; or due to the fact that patients were considered to be of low risk [(NPI) ≤ 3.4]. High risk (NPI > 3.4) patients (*n* = 351) received adjuvant chemotherapy; 149 patients (treated before 2000) received CMF, (cyclophosphamide, methotrexate and 5-fluorouracil), whereas 202 patients (treated after 2000) received anthracycline-combination adjuvant chemotherapy (Supplementary Table S1) [5]. 503 cases did not receive any chemotherapy, either because the patient declined systemic treatment, adjuvant chemotherapy was not the standard of care at that time, or patients were of low risk [Nottingham Prognostic Index (NPI) ≤ 3.4]. All patients were consented as per hospital Standard of Care. The study was approved by Nottingham Ethics Committee (C202313) and the Hospital Research and Innovations Department.

### Tissue microarray (TMA) and immunohistochemistry (IHC)

The TMAs were constructed and immunohistochemically profiled for pol β, FEN1, APE1, XRCC1, SMUG1, PARP1, ATR, ATM, Chk1, Chk2, p53, BRCA1, DNA-PKcs and TOPO2 (Supplementary Table S2). We have reported the specificity of the antibodies used here in recent previous publications [5, 23, 29–35]. Expression of HER2, ER and PR was re-assessed according to the American Society of Clinical Oncology/College of American Pathologists (ASCO/CAP) guidelines [36, 37]. For ER status, the EP1 clone has been used (Dako-Cytomation). ER and PR assays were considered negative if there were < 1% positive tumour nuclei in the presence of the expected reactivity of internal and external controls.

Tumour cores were evaluated by two pathologists (co-authors: TAF, IOE) who were blinded to the clinico-pathological characteristics of patients in two different settings. Whole field inspection of the core was scored and intensities of nuclear staining were grouped as follows: 0 = no staining, 1 = weak staining, 2 = moderate staining, 3 = strong staining. The percentage of each category was estimated (0–100%). H-score (range 0–300) was calculated by multiplying intensity of staining and percentage staining. H-score cut-offs for individual marker is summarized in Supplementary Table S2. Not all cores within the TMA were suitable for IHC analysis as some cores were missing or lacked tumour. Intra- (kappa > 0.8; Cohen kappa test) and inter- (kappa > 0.8; using multi-rater kappa tests) observer agreements were excellent. In cases where discordant results were obtained, the slides were re-evaluated by both pathologists together and a consensus was reached.

To validate the use of TMAs for immuno-phenotyping, full-face sections of 40 cases were stained and protein expression levels were compared. The concordance between TMAs and full-face sections was excellent (*k* = 0.8). Positive and negative (by omission of the primary antibody and IgG-matched serum) controls were included in each run.

### Calculation of DNA repair prognostic index scores

After definition of factors that were associated with BCSS in univariate analysis, multivariate Cox proportional hazards models (with backward stepwise exclusion of these factors, using a criterion of *p* < 0.05 for retention of factors in the model) were used to identify factors that were independently associated with clinical outcomes. The statistical significance of the model was assessed based on the likelihood ratio test. The proportional hazards assumption was tested using both standard log-log plots and by generating Kaplan–Meier survival estimate curves, and observing that the curves did not intersect with each other. Hazard ratios (HRs) for death risks and relapse and 95% confidence intervals were calculated from the Cox proportional hazards analysis. Subsequently, DNA repair index prognostic (DRPI) scores for BCSS were calculated using the summations of β-coefficient values of the factors retained in the final model after controlling for chemotherapies and lymph node stage.

### Determination of DNA repair prognostic index cut-offs

While DRPI score is a continuous risk, to evaluate its efficiency as a prognostic tool we defined subgroups associated with a prognostic outcome using specific cut-offs. We determined thresholds to define two DNA repair prognostic groups (DRPI-PGs) with distinct prognosis. To determine the cut-off point, a multivariate Cox regression model was used that included the clinical and demographic covariates and a dichotomous DRPI score based on cut-off points selected between the 5% and the 95% quantiles of the DNA repair prognostic index score distribution. The optimal cut-off point was selected as the quantile that maximized the profile log-likelihood of this model.

### Predictive accuracy of DNA repair prognostic index compared with other prognostic clinicopathological factors

To evaluate whether the DRPI adds new independent prognostic information to the lymph node stage, we performed separate Kaplan-Meier analyses by DRPI-PG within each LN stage stratum. The significance of the additional stratification provided by the DNA repair prognostic index was evaluated based on the log-rank test.

### Development and calculation of integrative prognostic index (IPI) score for ER negative BC

The LN stage score (1–3) has been added to DRPI scores to get an IPI continuous score (range = 3.39–0.09) and two IPI prognostic groups (IPI-PGs) were identified; IPI-PG1 = 3.39 to 1.09 and IPI-PG2 = 1.0 to 0.09. The thresholds to define the two IPI-PGs were selected by using a multivariate Cox regression model including the clinical and demographic covariates and a dichotomous IPI score. The optimal cut-off point was selected as the quantile of the IPI score that maximized the profile log-likelihood of the model.

### Clinical impact of DNA repair prognostic index and model discrimination

The receiver operating characteristic (ROC) curves were generated to compare the different prognostic models with and without inclusion of DRPI score [38]. Logistic fit of low vs. high survival category by cumulative hazard (the product of the hazard ratios of each incorporated variable) was performed. Area under the curve (AUC) value was calculated from the ROC curves. An AUC of 0.6 or above was considered a fair classifier. Model discrimination was evaluated based on Harrell's concordance index, or *c* index, which is a generalized area under the receiver operating curve (AUC) for censored observations and is equal to the probability of concordance between the predicted probability of relapse and the relapse outcomes. The concordance index was adjusted for bias using bootstrap resampling with 300 replications. The CI for the *c* index was obtained based on approximate normality using the variance estimate of the unadjusted index.

### Statistical analysis

Statistical analyses were performed using STATISTICA (Stat Soft Ltd, Tulsa, USA) and SPSS (version 17, Chicago, USA). Where appropriate, Pearson's chi-squared; student's *t*-test and ANOVA tests were used. All tests were two-sided with a 95% confidence interval (CI) and a *p* value of less than 0.05 was considered to be indicative of statistical significance. Survival data including survival time, disease-free survival (DFS), and development of loco-regional and distant metastases (DM) were maintained on a prospective basis. BC specific survival (BCSS) was defined as the number of months from diagnosis to the occurrence BC-related death. DFS was defined as the number of months from time of surgery to the occurrence of recurrence or DM relapse. Survival was censored if the patient was still alive, lost to follow-up, or died from other causes. Cumulative survival probabilities and 5-year BCSS and DFS were estimated using the univariate Cox models and the Kaplan–Meier plot method where appropriate, and differences between survival rates were tested for significance using the log-rank test. A stringent *p* value < 0.01 was considered to indicate statistical significance for multiple comparisons. Tumor Marker Prognostic Studies (REMARK) criteria, recommended by McShane et al [39], were followed throughout this study.

## SUPPLEMENTARY TABLES



## Abbreviations

ER: oestrogen receptor
PR: progesterone receptor
BER: base excision repair
TNBC: triple negative breast cancer
BRCA1: breast cancer susceptibility gene 1
DSB: double strand break
NER: nucleotide excision repair
polβ: DNA polymerase β
APE1: apurinic/apyrimidinic endonuclease 1
FEN1: flap endonuclease 1
XRCC1: x-ray cross complementation group 1
ATM: ataxia –telengiectasia kinase
ATR: ataxia-telengiectasia related kinase
DNA-PKcs: DNA dependent protein kinase catalytic sub-unit
TMA: tissue microarray
IHC: immunohistochemistry
DRPI: DNA repair prognostic index
DRPI-PGs: DNA repair prognostic index prognostic groups
IPI: integrated prognostic index
IPI-PGs: integrated prognostic index prognostic groups
ROC: receiver operating characteristic
AUC: under the receiver operating curve
