# Supplementary material for: DNA repair prognostic index modelling reveals an essential role for base excision repair in influencing clinical outcomes in ER negative and triple negative breast cancers
Source: Oncotarget. 2015 Jun 1;6(26):21964–78. doi: 10.18632/oncotarget.4157 (PMC4673139; doi:10.18632/oncotarget.4157)
Supplement: Supplementary file 1 [file oncotarget-06-21964-s001.pdf]

SUPPLEMENTARY TABLES

Supplementary Table S1. Clinicopathological characteristics of ER- breast cancer cohort

Variable	Cases (Valid %)
**Menopausal status**	
Pre-menopausal	352 (44.2)
postmenopausal	445 (50.6)
**Tumour Grade (NGS)**	
G1	17 (1.9)
G2	84 (9.6)
G3	777 (88.5)
**Lymph node stage**	
Negative	546 (62.2)
Positive	332 (37.7)
**Tumour size (cm)**	
T1 a + b (≤1.0)	79 (9.1)
T1 c (>1.0 -2.0)	381 (43.9)
T2 (>2.0-5)	369 (42.6)
T3 (>5)	38 (4.4)
**Tumour type**	
IDC-NST	678 (85.0)
Tubular	19 (2.4)
ILC	23 (2.8)
Medullary (typical/atypical)	42 (5.3)
Others	36 (4.5)
**Mitotic Index**	
M1	66 (7.6)
M2	119 (13.7)
M3	686 (78.8)
**Lymphovascular Invasion**	
Absent	444 (51.0)
Present	426 (49.0)
**Tumour Subgroups**	
Non Luminal Her2 Overexpression	185 (25.1)
Basal Like	421 (57.0)
ER-/HER2- (non basal)	132 (17.9)

(*Continued*)

Variable	Cases (Valid %)
**Basal Like Phenotype**	
No	319 (43.1)
Yes	421 (56.9)
**Triple Negativity**	
No	197 (22.4)
Yes	635 (72.2)
**HER2 Expression**	
Negative Expression	668 (78.3)
Overexpression	185 (21.7)
**Survival at 10 years**	
Alive	589 (68.3)
Dead from disease	273 (31.7)
**Recurrence**	
No recurrence	524 (60.2)
Recurrence	347 (39.8)
**DNA Index Subgroups**	
1	211 (41.9)
3	239 (58.1)
**PolB Expression**	
Low	421 (59.3)
High	289 (40.7)
**FEN1 (Cytoplasmic)**	
Low	420 (72.3)
High	161 (27.7)
**FEN1 (Nuclear)**	
Low	401 (69.0)
High	180 (31.0)
**APE1 Expression**	
Low	196 (60.1)
High	130 (39.9)
**XRCC1**	
Low	146 (21.9)
High	521 (78.1)
**SMUG1 Expression**	
Low	234 (38.1)

(*Continued*)

Variable	Cases (Valid %)
High	380 (61.9)
**PARP1**	
Low	417 (69.0)
High	187 (31.0)
**BRCA1**	
Low	383 (61.4)
High	241 (38.6)
**ATM**	
Low	369 (62.0)
High	226 (38.0)
**ATR**	
Low	282 (43.9)
High	360 (56.1)
**DNA-PKc**	
Low	127 (39.9)
High	191 (60.1)
**pChk1(Nuclear)**	
Low	694 (91.9)
High	61 (8.1)
**pChk1 (Cytoplasmic)**	
Low	210 (27.8)
High	545 (72.2)
**Chk2**	
Low	327 (55.2)
High	265 (44.8)
**p53**	
Low	333 (44.2)
High	420 (55.8)
**TOP2A**	
Low	329 (50.8)
High	319 (49.2)

NPI; Nottingham prognostic index, PG; prognostic group

Supplementary Table S2. Antigens, primary antibodies, clone, source, optimal dilution and scoring system used for each immunohistochemical marker

Antigen	Antibody	Clone	Source	Antigen Retrieval	Dilution / Incubation Time	Distribution	Scoring system	Cut-offs
**ER**	Mouse MAb anti-ER-α	SP1	Dako-Cytomation	Citrate pH6	1:150 30 min	Nuclear	Allred score	≥ 3 (positive)
**ER**	Mouse MAb anti-ER-α	EP1	Dako-Cytomation	Citrate pH6	1:80 30 min	Nuclear	% positive cells	≥ 1% positive
**PR**	Mouse MAb anti-PR	PgR636	Dako-Cytomation	Citrate pH6	1:125 30 min	Nuclear	% positive cells	≥ 1% positive
**HER2**	Rabbit antihuman c-erbB2	polyclonal	Dako-Cytomation	None	1:400 60 min	Membrane	See text	See text
**BRCA1**	BRCA1	MS110	Calbiochem	Citrate pH6	1:100 60 min	Nuclear	% of positive cells	< 25% (negative)
**ATM**	Rabbit MAb anti-ATM	Y170	Abcam	Citrate pH6	1:100 18 hours	Nuclear	% of positive cells	< 25% (negative)
**ATR**	Mouse MAb Anti-ATR	1E9	Novus Biologicals	Citrate pH6	1:20 18 hours	Nuclear	H-score	≥ 60 (High)
**Chk2**	Rabbit polyclonal anti-Chk2	Ab47433	Abcam	Citrate pH6	1:100 60 min	Nuclear	H-score	≥ 100 (High)
**pChk1**	Rabbit anti-pChk1	Ab58567	Abcam	Citrate pH6	1:140 60 min	Nuclear	H-score	≥ 50 (High)
**DNA-PKcs**	Mouse MAb Anti-	3H6	Abcam	Citrate pH6	1:1000 20min	Nuclear	H-score	> 260 (high)
**PARP1**	Mouse MAb Anti-PARP1	7D3-6	BD pharmingen	Citrate pH6	1:1000	Nuclear	% of positive cells	≥ 10% (positive)
**SMUG1**	goat MAb anti-SMUG1		Acris Antibody GmbH	Citrate pH6	1/200 15 min	Nuclear	H-score	> 35 (positive)
**APE1**	Rabbit polyclonal anti-APE1	NB100-101	Novus Biologicals	Citrate pH6	1:500 60 min	Nuclear	H-score	≥ 100 (positive)
**XRCC1**	Mouse MAb Anti-XRCC1	33-2-5	Thermo-scientific	Citrate pH6	1:200 20 min	Nuclear	% of positive cells	≥10% (positive)

(*Continued*)

Antigen	Antibody	Clone	Source	Antigen Retrieval	Dilution / Incubation Time	Distribution	Scoring system	Cut-offs
**FEN1**	rabbit polyclonal anti-FEN1	NBP1-67924	Novus Biologicals	Citrate pH6	1:200 15 min	Nuclear	H-score	≥ 100 (positive)
**TOP2A**	Mouse MAb	KiS1	Dako-Cytomation	Citrate pH6	1:150	Nuclear/ cytoplasmic	% of positive cells	> 25% (positive)
**p53**	Mouse MAb anti p53	DO7	Novocastra	Citrate pH6	1: 50 60 min	Nuclear	% of positive cells	≤ 20% (negative) > 20% (High)
**Ki67**	Mouse MAb anti-Ki-67	MIB1	Dako-Cytomation	Citrate pH6	1:300	Nuclear	% of positive cells	< 10% (low) 10–30% (moderate) > 30% (high)
